# Danysz's Microbe

**Published:** 1901-04-06

**Authors:** 


### DANYSZ'S MICROBE.

The discovery of a microbe which proved fatal to rats, coupled with the belief that rats usually devoured the bodies of their dead friends, has led to the hope that it might be possible, by infecting rats with this fatal microbe, and turning them loose among their fellows, to set up a rat epidemic which should rid us of these disagreeable and (in view of their relation to plague) dangerous pests. Experiments, however, have recently been made with Danysz's microbe at Athens, which have shown that, at least in regard to the rats of the drains of Athens, the microbe is not sufficiently virulent to act effectively, while it has the further disadvantage that the survivors do not seem to devour the dead bodies of those which had been infected so that the malady failed to spread. The utility of the process may therefore be questioned.

